# Genetic and environmental influences on the size-fecundity relationship in *Aedes albopictus* (Diptera: Culicidae): Impacts on population growth estimates?

**DOI:** 10.1371/journal.pone.0201465

**Published:** 2018-08-02

**Authors:** Katie S. Costanzo, Katie M. Westby, Kim A. Medley

**Affiliations:** 1 Department of Biology, Canisius College, Buffalo, New York, United States of America; 2 Tyson Research Center, Washington University, St. Louis, Missouri, United States of America; CSIRO, AUSTRALIA

## Abstract

Population growth models are integral to ecological studies by providing estimates of population performance across space and time. Several models have been developed that estimate population growth through correlates of demographic traits, as measuring each parameter of the model can be prohibitive in experimental studies. Since differences in female size can accurately reflect changes in fecundity for many taxa, Livdahl and Sugihara developed a population growth index that incorporates size-fecundity relationships as a proxy for fecundity. To investigate the extent to which this model is robust to variation of this proxy, we tested if genetic (source population), temperature and resource treatments affect the size-fecundity relationship in *Aedes albopictus* (Skuse), the Asian tiger mosquito. We then determined if variation in the size-fecundity relationship alters the population growth estimates, lambda (λ*’*), when applied to Livdahl and Sugihara’s model. We performed 2 laboratory experiments in which we reared cohorts of four different geographic populations of *A*. *albopictus* across 5 temperature treatments (18, 21, 25, 18, 31°C) and three resource treatments (low, medium, high larval resources). We determined if the slope of the size-fecundity relationship varied by source population, temperature, or resource; and if variation in this relationship affects lambda (λ*’*) estimates in a competition study between *A*. *albopictus* and *Culex pipiens* (Linnaeus), the northern house mosquito. Temperature treatments significantly affected the size-fecundity relationship, resource level marginally affected the relationship, while source population had no effect. We found positive relationships between size and fecundity when mosquito larvae were reared at high temperatures and low resource levels but the relationship disappeared when mosquitoes were reared at a low temperature or with high levels of resources. The variation in the size-fecundity relationship produced from different temperatures resulted in statistically different lambda (λ*’*) estimates. However, these changes in lambda (λ*’*) did not alter the trends in the population performance across treatments or conclusions of the competition study. This study provides evidence that the population growth model is sensitive to variation in size-fecundity relationships and we recommend biologists apply the most compatible size-fecundity relationship to the models to obtain the most accurate estimates of population performance.

## Introduction

Population growth rate is widely used to measure a population’s performance and project its size under certain environmental conditions. Population growth rates can vary substantially across space, and studying such variation can inform our understanding of environmental and demographic factors influencing population dynamics and evolutionary trajectories. In experimental studies, direct measures of population growth rates are often challenging because measuring variables required for its calculation—survival, development rate to adulthood, and fecundity—for individuals in each experimental cohort can be prohibitive. Consequently, indices are often derived that utilize significant correlates of these demographic parameters as proxies.

To utilize such proxies, Livdahl and Sugihara developed a composite index of population growth that estimates the per capita rate of increase, *r*’ [1]. This model can be applied when fecundity and/or adult mortality data are not easily collected; e.g. in organisms with relatively higher early life stage mortality compared to later, or reproductive stage mortality (type III survivorship), and uses previously established size-fecundity relationships as a proxy for fecundity. In ectotherms, fecundity generally increases with body size [2,3], and differences in female size can accurately reflect differences in fecundity without measuring fecundity itself [4]. Hence, population growth estimates such as Livdahl and Sugihara’s *r*’ which utilizes fecundity correlates, like female size, are common and efficient proxies, and the resulting *r*’ estimate can accurately predict trends in *r* across different environmental conditions [5]. However, such models assume that size-fecundity relationships are fixed, or their variation has negligible effects on population growth estimates when applied to these models.

In this paper, we investigate the effects of genetic and environmental factors on the size-fecundity relationship in the mosquito, *Aedes albopictus* (Skuse) (Diptera: Culicidae), commonly known as the Asian tiger mosquito. We then assess how changes in this relationship may affect the population growth estimate, λ’ of this species. Lambda (λ’) is a composite index of mosquito performance based on *r’*, which estimates the instantaneous population growth rate for each replicate. *Aedes albopictus* is a well-studied mosquito that serves as a common model system spanning diverse disciplines. It acts as an important vector of human disease such as dengue fever and chikungunya, and has been implicated as a potential vector for several other arboviruses including Zika [6–9]. This species is native to Asia and India [10], but has successfully invaded nearly every continent throughout the world including North America [11]. When introduced, *A*. *albopictus* often becomes widely established, and at times, is associated with a reduction of native resident mosquito populations [12–14], likely attributed to its superior competitive abilities during the larval stage [13,15,16]. Container-dwelling mosquitoes such as *A*. *albopictus* inhabit natural and artificial containers that retain water such as treeholes, tires, or basins during the immature larval stage, and reside among various microorganisms and invertebrates in these habitats. Because of their importance as an invasive species and to human health, along with the study of their container communities, *A*. *albopictus* serves as an excellent model for both ecological and medical studies. Regardless of the context, population growth is a common and useful variable estimated for this species to assess and predict its population performance across space and time.

In many studies involving mosquitoes, measuring fecundity of each individual female is prohibitive, as it involves successful mating, bloodfeeding, and oviposition for each individual female. Generally, positive linear relationships have been detected between mosquito female adult mass, pupal mass, or wing lengths and fecundity [4,17–21]. As such, many mosquito studies utilize Livdahl and Sugihara’s model to estimate *r’*, and do so by measuring female mosquito size or weight from their study and applying size-fecundity relationships obtained from other published studies [8,13,15,22–26]. Thus, the size-fecundity relationship is treated as being fixed. Variation exists in the size-fecundity relationship of other organisms across populations [27,28] and environments [29–31]. Although few studies have specifically evaluated plasticity in size-fecundity relationships in mosquitoes, there is evidence of genetically based variation in this relationship for *A*. *albopictus* across populations [32].

In this study, we extend these efforts by explicitly examining the role of genetic and environmental variation on the size-fecundity relationship in *A*. *albopictus*, and how different size-fecundity relationships affect the population performance index, λ’estimate [a derivative of Livdahl and Sugihara’s *r’*]. Although some studies incidentally evaluated the size-fecundity relationship in mosquitoes across environments [24], to date, no studies have explicitly investigated the effects of environmental conditions on the size-fecundity relationship in *A*. *albopictus*. We test four populations of *A*. *albopictus* from the United States to re-evaluate the role of a genetic source of variation on the size-fecundity relationship and investigate the role of temperature and larval resource levels on this relationship in these populations in two laboratory experiments. In this species, temperature [19,23,33], and resource limitation or crowding (density-dependent effects) [21,34] affect adult size and fecundity, but to our knowledge, it has not yet been evaluated if these factors affect the size-fecundity relationship. We predict that source populations along with temperature and resources will produce variation in size-fecundity relationships in *A*. *albopictus*. We also predict that significant differences in the size-fecundity relationship will result in different population growth estimates, λ’.

## Materials and methods

### Experiment 1: Size-fecundity variation by temperature

#### Larval and adult rearing

Experiments were conducted using F2 progeny of field-collected *A*. *albopictus* larvae from Beaufort, SC; Harrisburg, PA; Huntsville, AL; and Peoria, IL. Eggs were hatched in deionized (DI) water in tripour beakers with 0.35 g of nutrient broth (Difco Laboratories, Detroit, MI) per liter. Following hatching, 50, ~24 hour old first instar larvae were filtered from the hatching water, counted, and added to 400 ml tripour beakers. The beakers had 350 ml of DI water and 0.05 g of a 1:1 ratio by of brewer’s yeast:lactalbumin as a resource. They were placed in one of five environmental chambers representing each of the following five temperature treatments, 18, 21, 25, 28, and 31^o^ C, all with a 16:8 L:D photoperiod. Each temperature treatments had four replicates (beakers) of each population yielding a total of 80 experimental units. In the 21, 25, 28, and 31^o^ C treatments, 0.05 g resources were added to each replicate on day 5 and 0.03 grams were added on day 13 to prevent resource depletion. The 18^o^ C treatment experienced very slow rates of development and resource depletion (visually determined), thus 0.03 g of resources were only added to beakers of this treatment on day 30.

Each day, pupae from each replicate were transferred into vials with DI water until eclosion (emergence to adult). Newly eclosed adults were transferred daily into 1-L paperboard cages with mesh screening. Females from the same temperature treatment and replicate that emerged on the same day were housed in the same paperboard cage with up to 15 females per cage. To achieve 1:1 sex ratios in the adult cages, males that emerged within 5 days of females from the same temperature treatment and replicate were housed in the same paperboard cage with up to 30 adults total. Adults were housed in their respective temperature treatment with continuous access to a 10% sucrose solution. At day 5 following emergence, the 10% sugar solution was removed and adults were only given access to water to promote bloodfeeding. At day 7 following emergence, adults in cages were allowed access to a blood meal of citrated bovine blood (Hemostat Laboratories, Dixon, CA) with pig intestines as an artificial membrane. Bloodmeals were either administered using an artificial membrane feeder (Hemotek ®) or by heating the blood filled membranes with warm water. The females were allowed to feed to repletion and following bloodfeeding, engorged bloodfed females were separated and placed in 0.5-L paperboard cages with access to 10% sucrose solution in their respective temperature treatment. They were maintained for 5 days upon which they were sacrificed by freezing (-20^o^ C).

If females failed to bloodfeed on the first attempt (day 7) they remained in the large paperboard cage with males and were given access to 10% sucrose solution until day 8. On day 8, we replaced the sugar solution with water, and the females were provided an additional opportunity to blood feed on day10 post-emergence using the same procedures as above. Any females that bloodfed were processed as those that bloodfed on day 7. Any females that failed to bloodfeed during both attempts were sacrificed following the second attempt by freezing.

All bloodfed females were forced to retain their eggs and fecundity was estimated through ovary dissection. Female mosquitoes are known to retain mature eggs [35] especially under suboptimal conditions such as small laboratory cages. When experimentally estimating total fecundity, it is a common method to count both laid eggs and dissect ovaries to count mature follicles [36–40]. We simplified the process by counting only mature follicles in the ovaries as we were not specifically interested in the number of laid versus retained eggs. Bloodfed females were maintained in the freezer until processing. Prior to ovary dissection, the thorax (with wings) was removed and placed in a drying oven at 60^o^ C. The mosquito abdomen was then dissected to remove the ovaries and the number of mature follicles was counted to estimate fecundity. After the thorax remained in the drying oven for at least 48 hours, the wings were dissected and measured using ImageJ software.

### Experiment 2: Size-fecundity variation by resource

#### Larval and adult rearing

Experiments were conducted using F4 progeny of field-collected *A*. *albopictus* larvae from Beaufort, SC; Harrisburg, PA; Huntsville, AL; and Peoria, IL. Hatching protocol was identical to the first experiment, and 50, ~24-hour-old first instar larvae were added to a 400 ml tripour beaker. The beakers had 350 ml of DI water and one of the following resource level treatments of a 1:1 ratio by of brewer’s yeast:lactalbumin: Low: 0.001 g/larvae, Medium: 0.0025 g /larvae, or High: 0.004 g/larvae. The beakers were placed in environmental chambers at 25^o^ C with a 16:8 L:D photoperiod. There were 4 replicates of each population within each of the three resource treatments, yielding a total of 48 experimental units. Every two days, we placed the larvae in fresh DI water with the same resource concentration as the first day to account for larvae that died or pupated.

Each day, pupae were picked from beakers and newly eclosed adults were transferred daily into 1-L paperboard cages, same as the first experiment. Females from the same resource treatment and replicate that emerged on the same day were housed in the same paperboard cage with up to 8 females per cage. To achieve 1:1 sex ratios in the adult cages, males that emerged within 5 days of females from the same species, resource treatment, and replicate were housed in the same paperboard cage with up to 16 adults total. The total number of adults added to the large cages was reduced compared to the first experiment due to wing damage experienced by adults in the first experiment. Adults were housed at 25°C with a 16:8 L:D with continuous access to 10% sucrose solution. Identical bloodfeeding protocols as the first experiment were implemented on day 7 post-emergence, and day 10 post-emergence if they failed to bloodfeed on the first attempt. Following bloodfeeding, females were housed, processed, and wing length and fecundity were measured as in the first experiment.

To determine if there were any differences in the size-fecundity slopes across the temperature, resource treatments, or populations, we ran a separate Analysis of Covariance (ANCOVA) for each. Within each ANCOVA, fecundity (number of eggs) was the dependent variable and size (wing length) was the covariate (predictor). Depending on the experiment, either temperature or resource was the fixed factor (predictor) in the model. A third ANCOVA was run with population as a random factor (predictor) to determine if the size-fecundity relationship varied across populations. For this analysis, mosquitoes from both studies (temperature and resource) were pooled.

If differences in size-fecundity relationships across treatments (temperature, resource) or populations were illustrated, we determined if these differences resulted in different population growth estimates (λ’), a derivative of Livdahl and Sugihara’s *r’* model, which uses the size-fecundity relationship as a proxy for fecundity. To obtain the various size-fecundity relationships to apply to the model, we conducted separate linear regressions on the wing length (predictor) vs. fecundity (dependent) for each temperature and resource treatment, along with the combined source population data from both experiments. For regressions by each population, mosquitoes from both studies (temperature and resource) were combined. We also ran a linear regression on all mosquitoes combined across both experiments (now termed as the grand total mosquito populations).

#### Effects of size-fecundity relationships on population growth estimates from a competition study

Since we detected the greatest differences in the size-fecundity relationships across the temperature treatments (Tables 1 and 2), we selected these regression coefficients to incorporate into Livdahl and Sugihara’s *r’* model. Specifically, we applied these size-fecundity relationships to the model to estimate the performance of *A*. *albopictus* in a competition study between *A*. *albopictus* and the northern house mosquito, *Culex pipiens* L. that had been previously performed and published [41]. All other parameters of the model were derived from data collected from this previous study [41]. Our aim was to determine if variation in the size-fecundity relationship affect the population growth estimates, since constant size-fecundity relationships are often applied to the model.

**Table 1:** Temperature, resource, and population effects on the size-fecundity relationship.

Source	*DF*	*F*	*P*
Temperature	3, 356	3.92	0.0089
Resource	3, 384	3.97	0.0522
Population	3, 372	2.45	0.0621

Results of ANCOVA indicating if the slopes of the size-fecundity relationship vary across treatments or populations (size*treatment or population).

**Table 2:** Size-fecundity relationships across cohorts.

Source	Intercept ± SE	Slope ± SE	*Df* Model	*F*	*P*	*R*^*2*^
Grand Total	-94.72 ± 8.55	58.85 ± 3.06	1, 744	370.35	<0.0001	0.33
21^o^ C	116.52 ± 77.55	-12.44 ± 25.11	1, 49	0.23	0.6344	0.0047
25^o^ C	- 107.19 ± 23.51	62.71 ± 8.66	1, 129	52.47	<0.0001	0.29
28^o^ C	- 48.06 ± 59.71	40.57 ± 21.91	1, 33	3.43	0.0731	0.09
31^o^ C	- 67.55 ± 17.69	47.63 ± 6.89	1, 141	47.80	<0.0001	0.25
High	31.76 ± 47.80	15.86 ± 16.24	1, 90	0.95	0.3312	0.01
Medium	-94.25 ± 29.63	60.26 ± 10.15	1, 123	35.23	<0.0001	0.22
Low	-80.42 ± 28.82	54.09 ± 10.20	1, 167	28.14	<0.0001	0.14
Beaufort, SC	-121.49 ± 17.18	68.81 ± 6.23	1, 151	122.43	<0.0001	0.45
Harrisburg, PA	- 82.59 ± 14.99	54.71 ± 5.36	1, 194	103.98	<0.0001	0.35
Huntsville, AL	- 121.07 ± 20.3	69.66 ± 7/21	1, 177	93.23	<0.0001	0.35
Peoria, IL	- 76.27 ± 15.86	50.84 ± 5.65	1, 216	81.10	<0.0001	0.28

Summaries of separate regression equations predicting fecundity of *A*. *albopictus* from wing length (size) across temperature and resource treatments, and populations.

In this previous competition study, *A*. *albopictus* larvae were reared either alone or with *C*. *pipiens* larvae to assess the relative impact of intra- versus interspecific competition. Each competition treatment was crossed with 5 resource treatments which were percentages of Giant Foxtail grass (*Setaria faberi*) compared to senescent American elm leaves (*Ulmus americana*) as the larval resource (100%, 75%, 50%, 25% or 0% elm [a.k.a. 100% grass]). Giant Foxtail grass has a relatively higher rate of decay and faster release of nutrients compared to elm leaves; it was predicted that the competitive effects of *A*. *albopictus* on *C*. *pipiens* would be alleviated in treatments represented by a higher proportion of grass, as seen with other species [42]. One of the response variables measured across these treatments was lambda (λ’), a derivative of Livdahl and Sugihara’s *r’*, to estimate the instantaneous population growth rate for each replicate. The mosquito performance, (*r’*) for *A*. *albopictus* [1] was estimated for *A*. *albopictus* across each competition treatment (alone or with *C*. *pipiens*) and resource treatment (100%, 75%, 50%, 25% or 0% elm). From *r’*, the finite rate of increase or lambda (λ’) was calculated as = exp (*r*’). *r’* was calculated for each cohort as follows:

r′=[ln[(1N0)∑xAxf(wx)]D+[∑xxAxf(wx)∑xAxf(wx)]]

Where N_0_ is the initial number of females in a cohort (assumed to be 50% of the initial cohort), *A*_*x*_ in the number of females eclosing on day *x*, *w*_*x*_ is a measure of mean female size on day *x* per replicate, *f*(*w*_*x*_) is a function relating fecundity to female size, and D is the time (in days) for a newly eclosed female to mate, obtain a blood meal, and oviposit. For *A*. *albopictus*, D was assumed to be 14 days and the size-fecundity relationship used in this study was *f(w*_*x*_) = 78.02 *w*_*x*_ -121.240 (R^2^ = 0.173, N = 91, P < 0.0001) (derived from [43]), with *w*_*x*_ = wing length in mm. It is standard practice to apply a size-fecundity relationship obtained from different populations under different conditions to calculate lambda in a particular study. One of the objectives of the current study is to determine if applying the different size-fecundity relationships derived from our temperature treatments would result in different lambda (λ’) estimates, or different trends in lambda (λ’) across treatments in the competition study.

We incorporated the regression equation coefficients of the size-fecundity relationships across our temperature treatments from our current study into the lambda (λ’) calculations from the competition study as *f*(*w*_*x*_). All other parameters included in the new lambda (λ’) models were derived from data from the previous study [41]. Thus, the only parameter we changed in the new analyses was the function *f*(*w*_*x*_), which is generally treated as insensitive to changes in environmental conditions. To determine whether different size-fecundity *f*(*w*_*x*_) relationships produced by various temperatures altered lambda (λ’) estimates, we ran an Analysis of Variance (ANOVA) on the lambda (λ’) estimates with source of size-fecundity relationship, competition treatment (alone or with *C*. *pipiens*), percent elm resource treatments (100%, 75%, 50%, 25% or 0% elm), and the interactions as fixed effects. To determine if the size-fecundity relationships produced by different temperatures affected the trends in lambda (λ’) across competition and percent elm treatments, or the conclusions of the previous study, we ran an ANOVA on the lambda (λ’) estimates derived from these relationships with competition and percent elm treatments as fixed factors. Follow-up Tukey tests (α = 0.05) were performed to detect any pairwise differences between lambda (λ’) of *A*. *albopictus* across source of size-fecundity relationships, competition and percent elm treatments. All results could then be compared to those from the original study.

## Results

**Fig 1:**
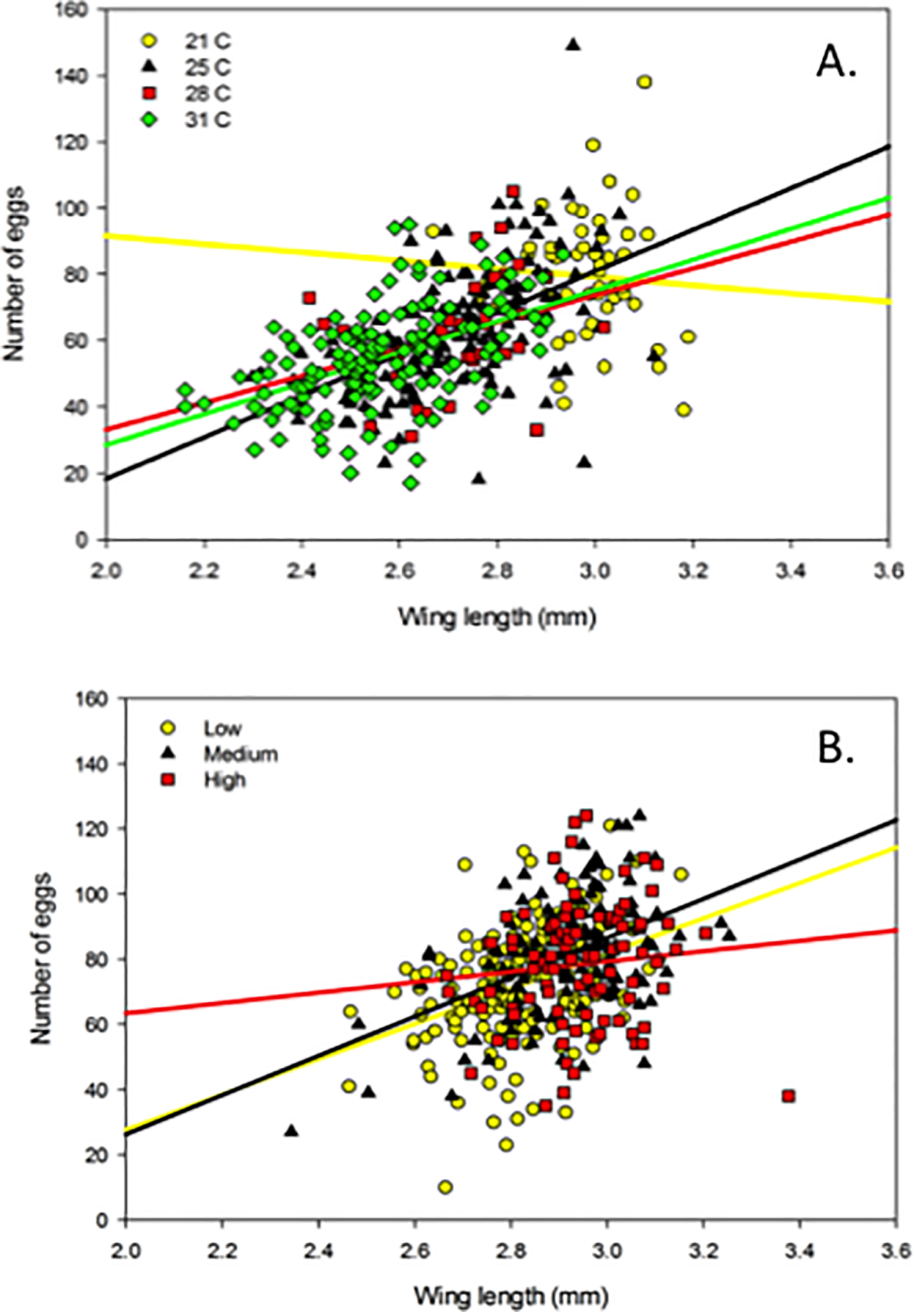
Size-fecundity relationships across temperature and resource treatments. (A) Size-fecundity relationships of *A*. *albopictus* across temperature treatments (N = 360). (B) Size-fecundity relationships of *A*. *albopictus* across resource treatments (N = 386).

**Fig 2:**
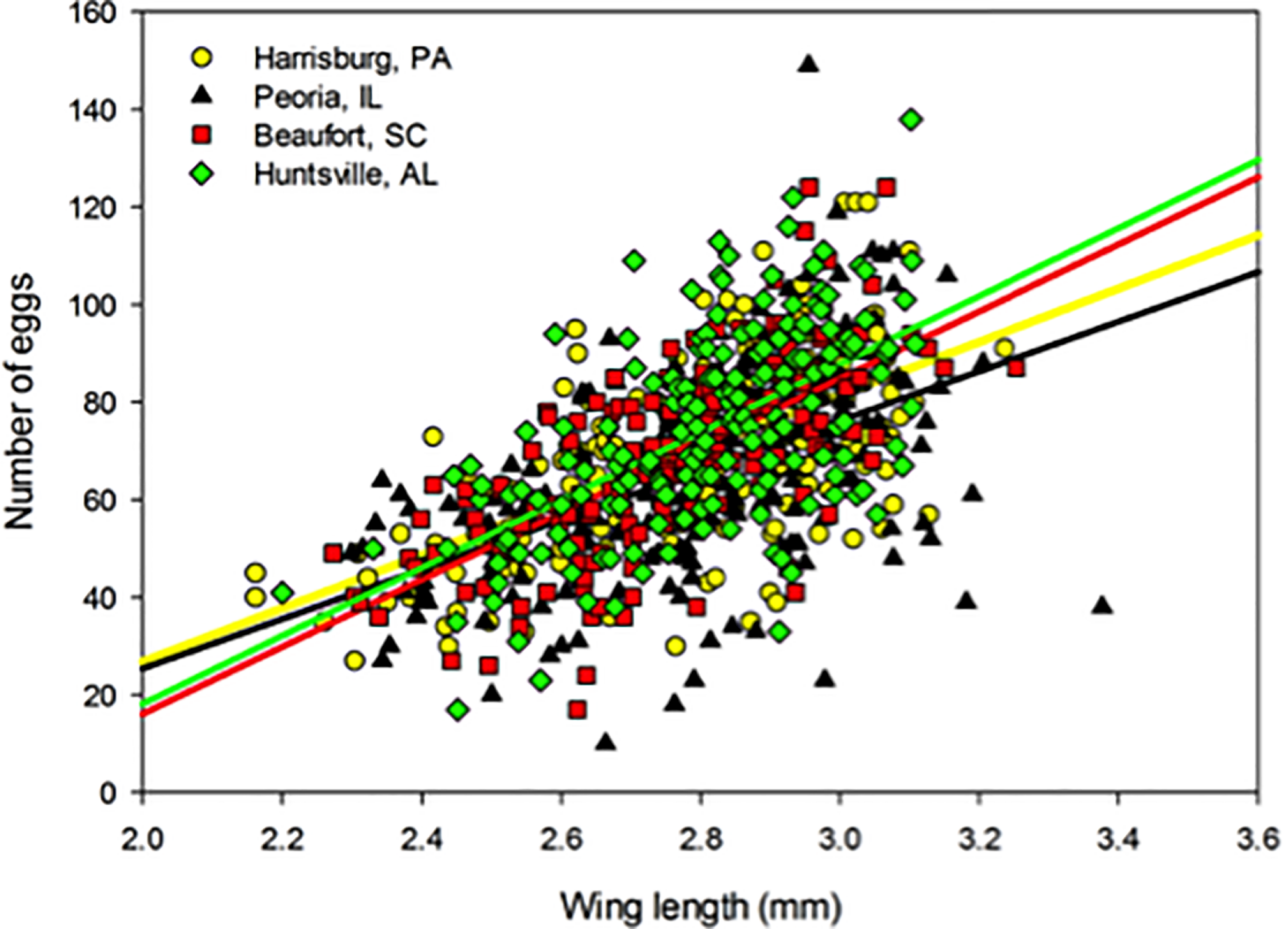
Size-fecundity relationships across populations. Size-fecundity relationships of *A*. *albopictus* across populations of all mosquitoes pooled together from both experiments (N = 746).

Due to low larval survivorship (0.2 ± SE 0.07) and few emerged adult females, we omitted data from the 18°C treatment for the temperature experiment. The ANCOVAs indicated that the slopes of the size-fecundity relationships varied across temperature, while resource marginally affected this relationship, and source population had no effect (Table 1, Figs 1A, 1B and 2). The regression coefficients and R^2^ values obtained from the size-fecundity relationships of all mosquitoes combined (grand total), temperature and resource treatments, and across populations from both studies illustrated variability in the size-fecundity relationships both among and within mosquito cohorts (Table 2, Figs 1 and 2).

**Fig 3:**
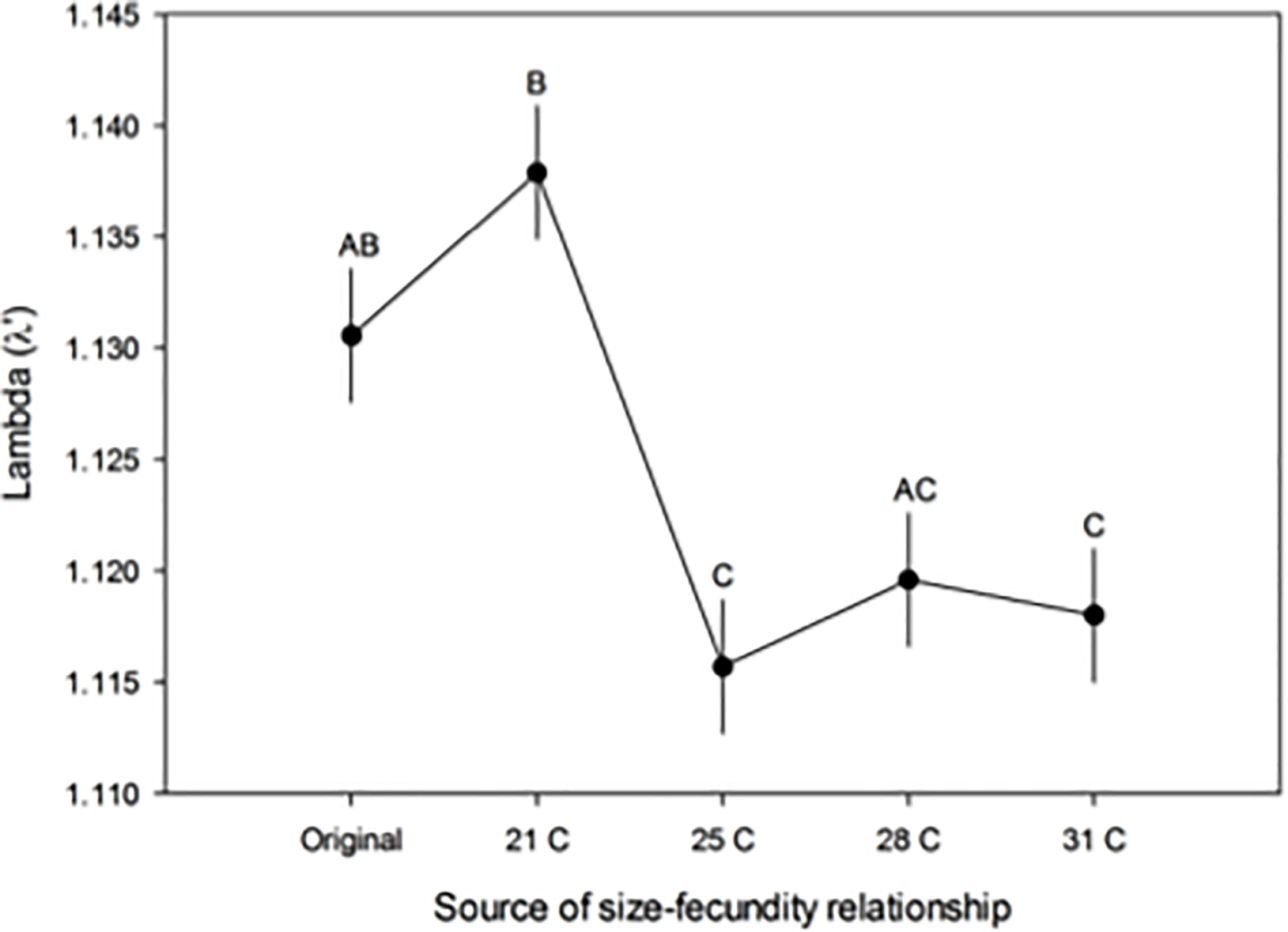
Effect of size-fecundity relationships on population growth estimates. Mean lambda (λ’) estimates (± 1 standard error) for *A*. *albopictus* using the size-fecundity relationship *f*(*w*_*x*_) applied in the previously published study [41] and those obtained across temperature treatments in the current study. Different capital letters indicate different pairwise comparisons between the lambda (λ’) estimates derived from different size-fecundity relationships.

The size-fecundity relationships derived from the temperature treatments significantly affected *A*. *albopictus* lambda (λ’) estimates from the previously published competition study (F_4, 201_ = 10.65, P < 0.0001). Overall, the lambda (λ’) estimates derived from the size-fecundity relationship obtained from mosquitoes reared at 21°C yielded significantly higher lambda (λ’) estimates than those derived from mosquitoes reared at 25, 28 and 31°C, which were not statistically different from the original estimates (Fig 3). The interactions between source of the size-fecundity relationship, competition treatment, and percent elm were not significant.

**Fig 4:**
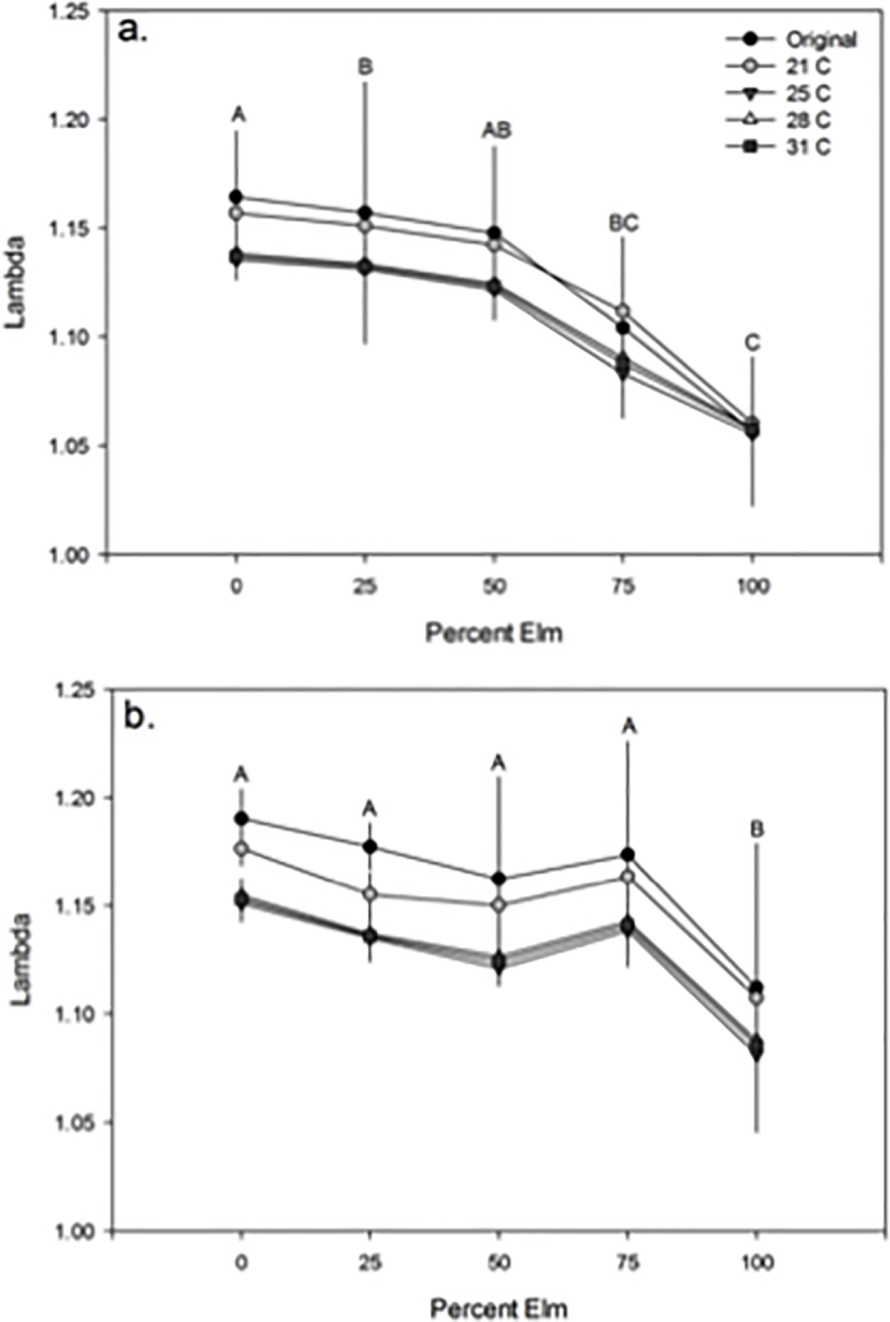
Effects of size-fecundity relationships on population growth trends. Mean lambda (λ’) estimates (± 1 standard error) for *A*. *albopictus* using the size-fecundity relationship *f*(*w*_*x*_) applied in the original study [41] and those acquired across temperature treatments in the current study. Estimates are illustrated across percent elm treatments (100%, 75%, 50%, 25% or 0% elm) in either the competition treatment with *A*. *albopictus* occurring alone (a), or with *C*. *pipiens* (b). Different capital letters indicate different pairwise comparisons of λ’ across percent elm treatments within each competition treatment.

Although the various size-fecundity relationships derived from rearing *A*. *albopictus* across different temperatures significantly affected the lambda (λ’) estimates, the trends in (λ’) estimates in the competition experiment remained consistent. Regardless of which size-fecundity relationship was applied, the ANOVAs on the effect of competition and percent elm treatments on lambda (λ’) revealed a consistent significant effect of treatment (alone or with *C*. *pipiens*), percent elm (100%, 75%, 50%, 25% or 0% elm), and interaction of competition and percent elm, as found in the original study. Furthermore, the paired comparisons between (λ’) estimates across treatments were identical to those in the original study when applying our size-fecundity relationships *f*(*w*_*x*_) in the model (Fig 4).

## Discussion

To our knowledge, this is the first study to specifically investigate how various environmental conditions (temperature and resource levels) along with population source affect the slope of the size-fecundity relationship in mosquitoes, an important model system across several disciplines including ecology and medical entomology. We found evidence for environmentally induced variation in the size-fecundity relationship in *A*. *albopictus*, and these differences in the size-fecundity relationships affect estimates of population performance through lambda (λ’). Overall, we found a consistent positive relationship between size and fecundity across most cohorts as identified in several studies [4,20,43]. However, we detected variability in this relationship; some populations and treatments exhibited strong, positive linear relationships, while some had no significant relationship between size and fecundity. Regarding genetic and environmental influences to variation in this relationship, our results support our prediction that the size-fecundity relationship for *A*. *albopictus* is affected by temperature.

Although theoretically, size-fecundity relationships are often considered fixed or insensitive to environmental gradients [44], our study together with others indicate that temperature can affect this relationship. In other organisms, steeper size-fecundity relationships have been detected in lower compared to higher temperatures [31,45–47]. Under higher temperature conditions, females invest less in reproduction compared to their similarly sized counterparts in lower temperatures; this has been explained through several mechanisms including trade-offs of higher growth rates, and greater maintenance costs/metabolic rates associated with higher temperatures [48–50]. In our study, we found opposite trends, with the shallowest size-fecundity relationship (flat) observed at the lowest temperature (21°C), while higher temperatures yielded significant, or nearly significant, positive relationships (Table 2, Fig 1A).

The majority of mosquito species require a bloodmeal to provision and mature eggs [51], and bloodmeal size and host type can affect fecundity [18,52]. However, reproductive output is also highly influenced by conditions experienced in the larval habitat. Adult fecundity is partially determined by total larval resource intake, which is affected by the quality and quantity of resources available along with the duration of larval feeding [18,53–55]. Teneral lipid reserves derived from the larval environments are used to provision eggs and affect reproductive output in the first and subsequent gonotrophic cycle [18,56–58]. Therefore, environmental conditions promoting greater lipid reserves in newly emerged females should result in proportionally greater reproductive output.

Such conditions that can lead to high teneral reserves include lower temperatures, in which mosquitoes generally take longer to develop into adults [51]. This slower development provides a longer duration of larval feeding, which translates into larger body size and greater surplus teneral lipid reserves in emerged adults [19,59]. Specifically, when larvae of *A*. *albopictus* were reared under a range of temperatures, lower temperatures (17^o^ C) produced adults with lipid reserves that increased exponentially with body size; i.e. small increases in body size were associated with relatively large increases in lipid reserves. When reared under higher temperatures (27 and 32 ^o^C), the larvae developed more quickly, had less time to feed, and illustrated a linear relationship of teneral lipid reserves, where lipid reserves were proportional to body size [19].

The effect of temperatures on developmental rate, larval feeding time, and lipid reserves is a reasonable explanation for the variation in the size-fecundity relationships across treatments in our study. Our higher temperature treatments (25, 28, and 31°C) produced positive relationships between size and fecundity, while the lower temperature treatment (21°C) yielded no relationship. The females in our lower temperature treatment also had longer development times (mean days to eclosion ± 1 standard error 21°C:16.31 ± 0.38; 25°C: 14.04 ± 0.24; 28°C: 10.04 ± 0.44; 31°C: 10.92 ± 0.21), providing more time for larval feeding, resulting in larger females (mean wing length mm ± 1 standard error 21°C: 2.98 ± 0.02; 25°C: 2.72 ± 0.01; 28°C: 2.72 ± 0.01; 31°C: 2.56 ± 0.01). As a result, females from the lower temperature likely reached the size increments with proportionally greater lipid reserves, diminishing the relationship between size and fecundity in this treatment. In contrast, in higher temperatures with faster development time and less time to feed, adult mosquitoes likely emerged with relatively lower teneral lipid stores. Therefore, under these conditions, one would expect to observe a stronger relationship between female size and fecundity, where lipid stores available for investment in egg production are proportional to body size increments.

The same pattern was observed for the congener *Aedes triseriatus* (Say), with no relationship between size and fecundity when mosquitoes were reared at lower temperatures, whether the temperature remained constant or fluctuated, and a positive relationship when reared under higher temperatures [24]. A flat size-fecundity relationship was also observed in older (17–15 days) *Culex quinquefasciatus* Say mosquitoes while younger females (5–13 days) exhibited a positive relationship [38], suggesting older females may have exhausted their teneral reserves removing the effect of adult size on fecundity.

Although previous studies illustrate variable effects of resource levels on fecundity [29,45,50,60], we found marginally significant effect of resource on the size-fecundity relationship (Fig 1B), with the highest resource treatment yielding no relationship (flat), but the medium and low resources yielding a positive relationship (Table 1, Fig 1B). The trends in our study across resource treatments may also be explained by different teneral lipid reserves acquired under different larval conditions.

In mosquitoes, high resource conditions during the larval stages also produce greater lipid reserves relative to size in emerging adults [55]. When *Aedes aegypti* (Linnaeus) mosquito larvae were reared across various resource levels, the larger adults emerging from higher resource conditions also illustrated an exponential increase in lipid stores relative to body size [18]. Additionally, the fecundity to body size ratio was greater in larger *A*. *aegypti* adults compared to smaller *A*. *aegypti* adults (fecundity:body size ratio: small: 10.8; medium: 17.93; large: 26.47; when fed human blood) [18] suggesting proportionally greater investment in egg production in the larger mosquitoes. In our study, the lack of relationship in size-fecundity in the High resource treatments is likely due to proportionally greater lipid stores built up under these resource conditions, reducing the relationship of fecundity on body size.

Interestingly, we found no significant effect of population source on the size-fecundity relationship (Fig 2). Although distinct *A*. *albopictus* populations have demonstrated different size-fecundity relationships, these populations of interest were under known differences in selection pressures important to the size-fecundity relationship [21,32]. In our study using populations from as much as ~1200 km apart, the effects of selection and drift did not result in differential resource allocation to fecundity among the four populations; they all yielded a strong, positive size-fecundity relationship (Table 2, Fig 2). This suggests that the size-fecundity relationship is robust to geographic variation, and more sensitive to local conditions under which larvae develop.

Our study illustrates that larval conditions can influence the relationship between body size and fecundity. In addition, the variation found in the size-fecundity relationships produced across temperature treatments significantly affected the population growth rates estimated by lambda (λ’). The size-fecundity relationships produced when *A*. *albopictus* was reared at 21°C resulted in significantly greater lambda (λ’) estimates compared to lambda (λ’) estimates applying the size-fecundity relationships from the 25, 28 and 31°C treatments (Fig 3). The temperature treatments that produced smaller mosquitoes, yielding steeper size-fecundity relationships with lower intercepts, consequently lowered the population growth estimate, (λ’). Therefore, our study suggests that different size-fecundity relationships can affect population growth rate estimates when applied to Livdahl and Sugihara’s *r’* model. While different size-fecundity relationships affected lambda (λ’) estimates, these changes did not alter the overall trends in lambda (λ’) in the previously published competition study (Fig 4). In other words, the conclusions of the competition study of *A*. *albopictus* lambda (λ’) were identical to the original findings [41], regardless of which size-fecundity relationship *f*(*w*_*x*_) was incorporated into the model. Other studies similarly found genetically based or hypothetical changes in the size-fecundity relationship do not alter the conclusions of population performance across treatments in competition studies [32,61].

In the competition study, *A*. *albopictus* lambda (λ’) estimates were greater than 1 across all treatment combinations, indicating positive population growth. However, in studies in which lambda (λ’) estimates are near or at 1, or zero population growth, slight changes in population growth rate due to variation in the size-fecundity relationships could substantially alter the conclusions of the study by yielding zero, positive, or negative population growth rates under the same experimental conditions. Thus, selection of the most appropriate size-fecundity relationship is imperative. If possible, we recommend one apply a size-fecundity relationship that is produced in environments compatible with those of their experiment in order to achieve the most accurate estimates of population growth rates. This is most important when the population growth rate estimates are near zero. Our study, for example, provides size-fecundity relationships for *A*. *albopictus* across various temperature and resource treatments that can be applied to future studies applying similar conditions.

The population growth rates of any population may be affected by survival, fecundity, and developmental rate to reproductive age; and the contributions of these demographic parameters on population growth have long been evaluated and debated [62–66]. Much theory predicts greater contributions of developmental rate and survivorship to population growth than fecundity [67–72]. In populations characterized by high fecundity and high population growth rates relative to survival rates such as mosquitoes, fecundity should contribute relatively little to population growth [51,70,71]. Despite this, we found that temperature treatments produced enough change in the size-fecundity relationship to significantly affect the population growth estimate. Population growth indices are integral to ecological studies that enable biologists to estimate population performance under various conditions. The development of models such as Livdahl and Sugihara’s *r’* that use proxies for demographic parameters have been a valuable tool for experimental studies and will continue to be in the future. Although these models are tremendously useful to biologists, the assumptions that size-fecundity relationships are fixed, or variation in this relationship is inconsequential to population growth, should be taken with caution. Our study suggests that in mosquitoes, size-fecundity relationships are affected by larval conditions and population growth estimates are sensitive to these changes. We postulate that larval conditions that promote proportionally greater lipid reserves (e.g. environments with greater overall resource intake) reduce the relationship of fecundity on size. When performing experimental studies, one should apply the most appropriate size-fecundity relationships to these models to yield the most accurate estimates of population performance. The range of populations and environmental conditions used in this study do not encompass all of the variation that mosquitoes experience under natural conditions, however we offer a useful starting point to begin incorporating more realistic estimates of fixed parameters (e.g. size-fecundity) into population growth models.

## Supporting information

S1 File DataSizeFecundityTemperatureStudy.csv. Data collected from study including wing legnth and number of eggs from each *Aedes albopictus* female that successfully bloodfed from the four populations across different temperatures.
(CSV)

S2 File DataSizeFecundityResourceStudy.csv. Data collected from study including wing legnth and number of eggs from each *Aedes albopictus* female that successfully bloodfed from the four populations across different resource levels.
(CSV)

S3 File DataLambda.csv. Population growth (lambda λ') estimates calculated for *Ae*. *albopictus* populations in the competition experiment when applying the size-fecundity relationships obtained from popoulations of Ae. albopictus reared across different temperatures.
(CSV)
